# A portable low-cost polymerase chain reaction device

**DOI:** 10.1016/j.ohx.2025.e00635

**Published:** 2025-02-25

**Authors:** Kan Luo, Wei Cheng, Yu Chen, Qirong Zhang, Chaobing Liang, Jianxing Li, Wu Wang

**Affiliations:** a School of Electronic, Electrical Engineering and Physics, Fujian University of Technology, Fuzhou 350118, China; b Fuzhou Industrial Integration Automation Technology Innovation Center, Fuzhou 350118, China; c School of Mechanical and Automotive Engineering, Fujian University of Technology, Fuzhou 350118, China; d College of Electrical Engineering and Automation, Fuzhou University, Fuzhou 350108, China

**Keywords:** Polymerase chain reaction (PCR), Temperature control, Arduino, PID control algorithm, Low-cost

## Abstract

Polymerase chain reaction (PCR) is a cornerstone technique in molecular biology and clinical diagnostics. However, conventional PCR systems are often bulky and prohibitively expensive, limiting their use in resource-limited settings. In this work, we present a portable, low-cost PCR instrument designed to overcome these challenges while providing fast and accurate thermal cycling. The system features a compact four-well aluminum heating block integrated with a semiconductor thermoelectric cooler and a heated lid, all controlled via an Arduino UNO platform and a piecewise variable coefficient PID algorithm. The device achieves heating and cooling rates of 1.78 °C/s and 1.52 °C/s, respectively, and maintains temperature accuracy within ± 0.55 °C. The power-bank powered prototype measures 210 × 140 × 105 mm^3^ and weighs 670 g, making it ideal for resource-constrained applications. Validation experiments, including successful amplification of kelp genes, yielded results comparable to conventional commercial instruments. Open source design files and detailed build instructions are provided under the MIT license, providing a cost-effective and accessible solution for expanding molecular diagnostic capabilities in resource-limited settings.

## Graphical abstract

**Figure:**
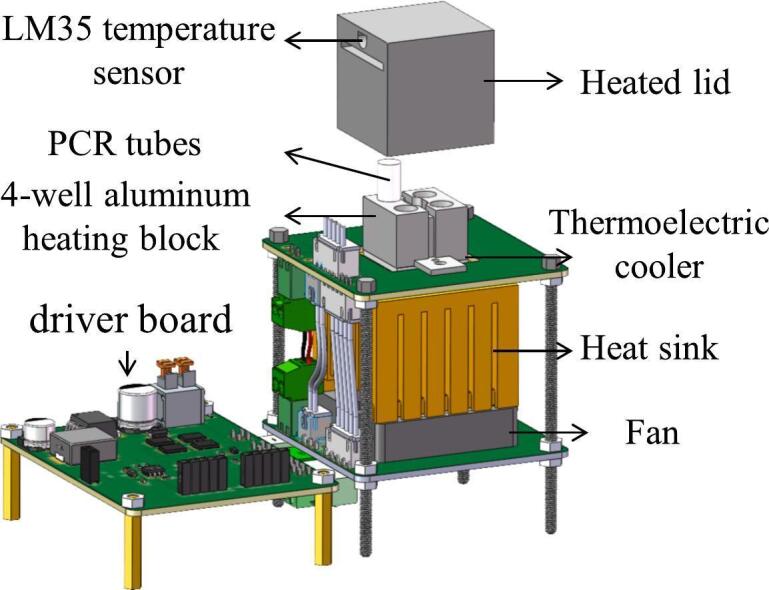

Specifications table

**Table:** 

Hardware name	A Portable Low-Cost Polymerase Chain Reaction Device
Subject area	• Engineering and materials science • Biological sciences (e.g., microbiology and biochemistry) • Educational tools and open source alternatives to existing infrastructure
Hardware type	• Measuring physical properties and in-lab sensors • Biological sample handling and preparation • Field measurements and sensors • Electrical engineering and computer science • Mechanical engineering and materials science
Closest commercial analog	Traditional commercial PCR instrument, such as ETC 811, Dongsheng (96 wells).But quite different from the proposed portable low cost 4 wells PCR device.
Open source license	MIT License
Cost of hardware	USD 120
Source file repository	https://zenodo.org/records/14875968

## 1 Hardware in context

Polymerase chain reaction (PCR) is a widely used technique for amplifying nucleic acids, making it essential for the quantitative analysis of DNA and mRNA, as well as for clinical diagnostics and pathogen detection [1], [2]. However, traditional commercial PCR thermal cyclers are typically expensive, bulky, and designed for use in laboratory settings. This restricts their applicability in resource-limited environments, particularly in developing regions and rural areas [3], [4]. These regions often face shortages of medical resources and difficulties in monitoring emerging epidemics, highlighting the pressing need for affordable, user-friendly diagnostic tools. Miniaturized and portable PCR devices could play a critical role in enabling rapid testing of small batch samples in such contexts [5], [6]. Therefore, there is an urgent need and great significance for the development of compact, highly integrated PCR devices.

PCR involves a cyclic process with three main steps, repeated over 20 – 40 cycles [7]. The first step, Denaturation, occurs at 94 – 98 °C, where the double-stranded DNA melts into single strands by breaking the hydrogen bonds between them. The second step, Annealing, involves lowering the temperature to 50 – 65 °C, allowing primers to bind to complementary sequences on the single-stranded DNA. These primers mark the specific region to be amplified. The third step, Extension, occurs at 72 °C, the optimal temperature for the heat-stable DNA polymerase enzyme (e.g., Taq polymerase), which synthesizes a new DNA strand by adding complementary nucleotides. Temperature control plays a critical role in the PCR process, as each step requires precise temperature settings to ensure accurate and efficient amplification [8]. Consequently, the temperature control unit is a key component of PCR, enabling the precise regulation of these temperature changes throughout the cycles.

PCR temperature control methods can be divided into two categories: contact and non-contact. The key distinction lies in whether the temperature control device physically contacts the PCR chamber [9]. Contact methods involve direct heat exchange with the PCR chamber. Early solutions, such as water bath heating [10], provided good temperature uniformity but were bulky and slow, making them unsuitable for portable applications. A more portable and cost-effective solution was introduced with electric wire heating using nickel–chromium (NiCr) wire heaters [11]. However, a more advanced method, semiconductor temperature control based on the Peltier effect [12], [13], has proven ideal for portable PCR devices. This method allows rapid switching between heating and cooling by adjusting the current direction in the semiconductor chip. It is compact, highly efficient, and easy to control, making it well-suited for the compact nature of portable PCR systems. Non-contact methods, such as light-wave radiation, electromagnetic induction, and chemical thermogenesis, do not require direct contact with the PCR chamber. Light-wave radiation heating [14], [15] uses near-infrared light to heat specific areas through gold nanostructures, which convert light into heat with high efficiency. Electromagnetic induction heating [16] generates heat by inducing eddy currents through an alternating magnetic field. Centrifugal air heating [17] uses hot air directed by a nozzle to achieve localized heating through convection. Chemical thermogenesis [18], [19] produces heat via chemical reactions, offering a high energy density and fast heating rate. However, these non-contact methods are hindered by challenges in rapidly switching between heating and cooling and by complexities in achieving precise temperature control, which has prevented their widespread adoption.

Parallel to the advances in temperature control, system integration remains a current research hotspot, with significant efforts directed toward developing low-cost, portable PCR systems. For example, Oliveira et al. [20] developed a low-cost PCR device using a custom PCB heater arranged in a sandwich-like configuration, providing a simple and economical solution. Although this device achieved a rapid heating rate of 2.0 °C/s, it suffered from limited temperature control precision and susceptibility to external interference. Similarly, Chong et al. [21] designed a portable real-time fluorescence quantitative PCR system with a 6-well, 0.1 mL reaction chamber that achieved temperature changes of up to 3.0 °C/s; however, the system's cost of around 2300 USD remains a barrier. Wu et al. [22] introduced a PCR method based on an oil bath in a pipeline configuration, which demonstrated good temperature uniformity but suffered from slow temperature transitions. Additionally, Kulkarni et al. [23] developed a microfluidic PCR device featuring serpentine microchannels fabricated via soft lithography. While this design significantly reduced the PCR amplification time, the high cost of microfabrication remains a drawback. Although progress has been made in enhancing portability and miniaturization, current PCR systems still encounter challenges in temperature control accuracy, high cost, or reliance on external power supplies. Balancing precise temperature control, cost efficiency, and miniaturization while maintaining overall performance remains a significant challenge in portable PCR system design.

In this study, we developed a low-cost, portable, and user-friendly PCR device utilizing a semiconductor thermoelectric cooler, specifically designed for use in resource-limited settings. Our work involved designing the PCR temperature control platform based on an Arduino UNO, along with the Peltier driver circuits, power supply module, and human–machine interface. The device is powered by a 120 W power bank, ensuring mobility and convenience. The prototype measures 210 × 140 × 105 mm^3^ and weighs 670 g. It achieved a heating rate of 1.78 °C/s and a cooling rate of 1.52 °C/s, with a total hardware cost of approximately 120 USD. The performance of our prototype was validated through PCR amplification in yeast two-hybrid assays, with a focus on heating and cooling rates, as well as steady-state temperature accuracy, ensuring precise and rapid thermal cycling. The experimental results aim to demonstrate the potential of our PCR device as a practical, affordable solution for PCR testing in resource-constrained environments.

## 2 Hardware description

**Fig. 1:**
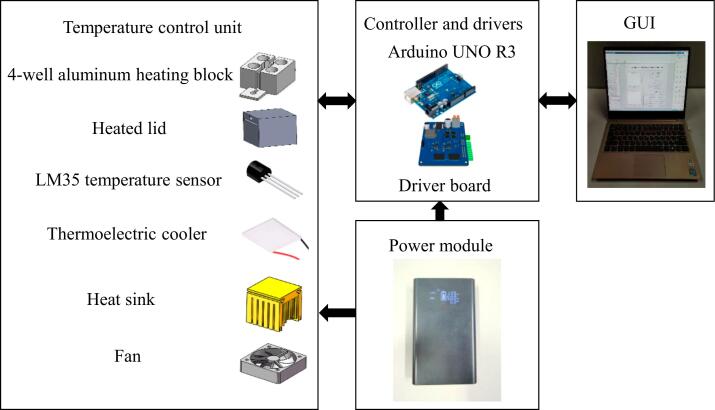
System framework diagram of our portable low-cost PCR device.

The system framework of our proposed miniaturized 4-well PCR device is shown in Fig. 1. It primarily consists of a temperature control unit, a controller and driver module, a power module, and a graphical user interface. The temperature control unit includes a 4-well aluminum heating block, a thermoelectric cooler, a heated lid, a heat sink, a high-speed fan, and LM35 temperature sensors. Temperature regulation is managed by an Arduino UNO board with an H-bridge drive circuit, while a MATLAB GUI on the PC serves as the user interface. Additionally, the system is powered by a 120 W power bank. In the following section, we will provide a detailed explanation of the device's main components, including the temperature control unit, Arduino UNO controller, and control software, etc.

### 2.1 Temperature control unit

**Fig. 2:**
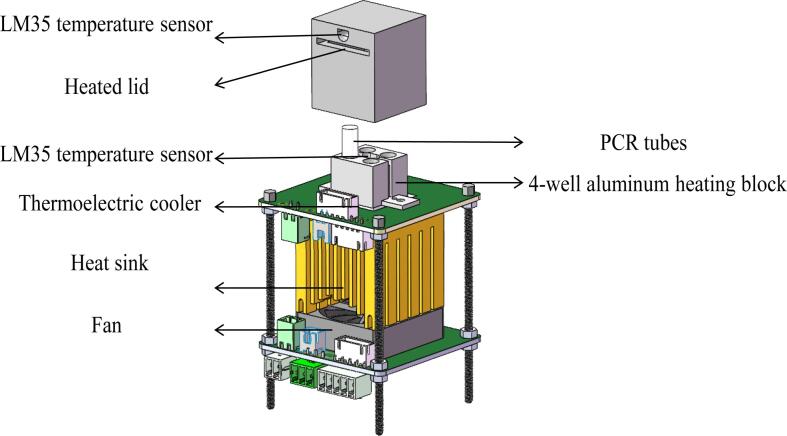
3D structure of the PCR temperature control unit.

The design of the temperature control unit is crucial to the functionality of the miniaturized 4-well PCR system. This unit is responsible for both heating and cooling the PCR samples, making it the core element of the thermal cycling process essential for PCR. The 3D structure of the PCR temperature control unit is shown in Fig. 2.

As shown in Fig. 2, the overall design emphasizes compactness and portability while ensuring reliable PCR amplification. The unit is composed of six main components arranged from top to bottom:

(1) Heated Lid: The heated lid (3D printed by Hangzhou Meiyi Orange Design Co., Ltd) assembly is designed to cover the tops of the PCR tubes, preventing condensation on the tube caps during the reaction by maintaining a higher temperature. It includes a slot for installing an LM35 temperature sensor to enable closed-loop control of the lid's temperature. The temperature is typically set to 105 °C but can be adjusted according to experimental requirements.
(2) 4-well Aluminum Heating Block: The block is made from aluminum alloy (3D printed by Hangzhou Meiyi Orange Design Co., Ltd), chosen for its good thermal conductivity, lightweight nature, and cost-effectiveness. It has dimensions of 20 mm × 20 mm × 14 mm and includes a 2 × 2 array of reaction slots that are designed to hold standard 0.2 mL PCR tubes. In the center of the block, a straight slot with a length of 3.18 mm and a height of 5.2 mm is provided for mounting an LM35 temperature sensor, allowing for precise closed-loop temperature control. The block also features two mounting ears, which are secured to the first PCB using M3 screws, ensuring stable installation.
(3) Thermoelectric Cooler: The system incorporates a TES1-12703 semiconductor thermoelectric cooler (Guangzhou Sirui Electronic Technology Co., Ltd.), measuring 20 mm × 20 mm × 1.65 mm, and operates at a maximum working power of 45 W with a 15.5 V voltage. One side of the cooler is tightly integrated with the bottom of the heating block, while the other side is attached to a heat sink, facilitating efficient thermal cycling. To ensure full contact and eliminate any air gaps caused by surface irregularities, thermal conductive silicone grease is applied on both sides of the thermoelectric cooler. The cooler’s wires are connected to terminal blocks on the PCB, which are then linked to the H-bridge driver circuit for precise temperature control.
(4) Heat Sink and High-Speed Fan: To enhance cooling efficiency, a fin heatsink (Frostwind computer coolong fan Co., Ltd) is used, which increases the surface area for heat dissipation through multiple vertical fins. Combined with active cooling from a high-speed fan (Shenzhen Qisu Electronic Technology Co., Ltd) rotating at 4,500 RPM, the heat is effectively transferred from the heat source and dispersed into the surrounding environment. The base of the heat sink is slightly larger than the 20 mm × 20 mm size of the TES1-12703 thermoelectric cooler to ensure proper fit. The combined heat sink and fan assembly must provide cooling capacity exceeding 45 W to ensure the thermoelectric cooler operates at maximum performance, consistently maintaining its full power output.
(5) LM35 Temperature Senors: The LM35 (Texas Instruments, USA) is a linear temperature sensor. It has an operating temperature range of − 55 °C to + 150 °C, provide accuracies of ± 0.75 °C. For this application, the LM35C variant is used, which has a measurement range of − 40 °C to + 110°C and a sensitivity of 10 mV/°C, providing an output voltage directly proportional to the temperature. The sensors are installed in the mounting holes on both the heated lid and the heating block to ensure effective temperature monitoring and control throughout the process.
(6) Hexagon Socket Head Cap Screws and Printed Circuit Boards: The structure adopts a modular design with two printed circuit boards (Shenzhen JLC Technology Group Co.,Ltd) connected by four hexagon socket head cap screws (Anqing Guji Hardware Co., Ltd). These pillars provide physical stability while the PCBs integrate part of the electrical circuitry, simplifying wiring and allowing for easy connections between components through plug-in mechanisms. This design enhances the overall portability and compactness of the system.

### 2.2 Controller and drivers

The Arduino UNO R3 [24] (Arduino Srl, Italy) was chosen as the microcontroller for our PCR system due to its cost-effectiveness and versatility. The 16 MHz clock speed of the Arduino UNO R3 provides precise timing control, which is essential for regulating temperature during the PCR process. When operating at 5 V, the board's 10-bit Analog-to-Digital Converter (ADC) achieves a voltage resolution of 4.88 mV per bit. This high-resolution measurement is crucial for accurate temperature monitoring when paired with the LM35 temperature sensor. The Arduino also features multiple PWM outputs, which are instrumental in managing the temperatures of different components: the H-bridge controls the 4-well aluminum heating block, while MOSFETs regulate the heated lid and the fan speed. Furthermore, the Arduino UNO R3 supports MATLAB programming, allowing for the implementation of advanced control and data processing algorithms using MATLAB's built-in functions. This integration significantly improves both the flexibility and the accuracy of the control system in our low-cost, open-source PCR setup.

Considering high-power components like the Peltier module, heated lid, and cooling fan consume significant energy compared to the controller. These components are powered directly through the drivers by an external high-power 15.5 V voltage source, such as a 120 W power bank. Then we designed a dedicated driver board that consolidates all the necessary driver circuits on one single PCB, called “driver board”. The board includes modules for driving high-power components and a voltage regulator to supply stable voltage to the driver chips, ensuring efficient and reliable system running.

**Fig. 3:**
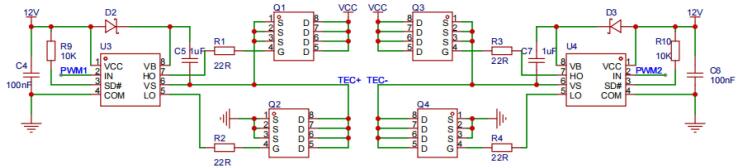
The schematic diagram of the H-bridge driving circuit for the Peltier module.

The H-bridge driving circuit for the Peltier module, as shown in Fig. 3, consists of four MOSFETs and a thermoelectric cooler (TEC) chip. The MOSFETs are controlled diagonally across the bridge arms to regulate the TEC's operation. When Q1 and Q4 conduct, current flows from the power supply's positive terminal through Q1, across the TEC, and returns via Q4 to the negative terminal. This causes the TEC’s top surface to heat and the bottom surface to cool. Conversely, when Q2 and Q3 conduct, the current flow reverses, switching the heating and cooling surfaces of the TEC. To ensure reliable operation and provide sufficient headroom above the TEC's operating parameters, N-channel MOSFETs (BSC0702LS, International Rectifier, USA) were selected, featuring a drain-source breakdown voltage of 60 V and a continuous drain current of 100 A. These specifications prevent damage to the system during operation. To avoid short-circuiting from simultaneous conduction of MOSFETs on the same bridge arm, a dead-time period is introduced between switching transitions. Two IR2104S (International Rectifier, USA) half-bridge driver chips are used to control each side of the H-bridge, with internal dead-time control ensuring safe switching. The PWM signals driving the H-bridge are configured with complementary outputs, preventing overlap between the high- and low-side switching. This design enhances the overall safety, stability, and efficiency of the system. It should be noted that a bootstrap circuit, consisting of capacitors (C5, C7) and Schottky diodes (D2, D3), provides the necessary voltage to drive the high-side MOSFETs. As the PWM control signals switch between on and off, the capacitors charge and discharge, ensuring that the half-bridge driver chips (U3, U4) operate under proper conditions throughout the process.

**Fig. 4:**
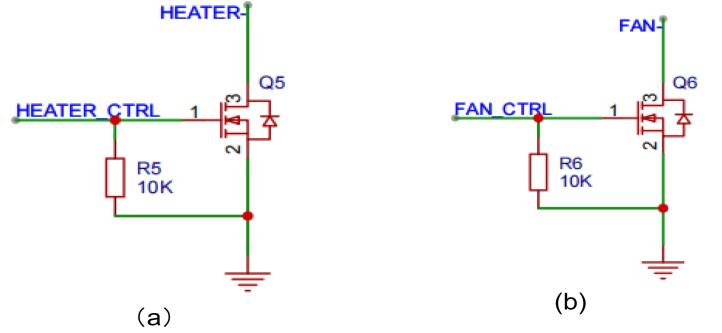
The schematic diagram of the N-channel MOSFET driver circuit for (a) the PTC ceramic of the heated lid and (b) the fan.

The fan and the heated lid driving circuit are shown in Fig. 4. Both circuits operate at a supply voltage of 12 V and are controlled using N-channel MOSFETs. Since the fan and heated lid are not frequently switched on and off during operation, and their operating currents are relatively low (0.26 A for the fan and 1.2 A for the heated lid), AO3400 MOSFETs(UMW, China) were chosen for these circuits due to their compact package. The MOSFETs can be controlled via the digital I/O ports of an Arduino UNO board with high and low voltage levels. Resistors R5 and R6 act as gate pull-down resistors for the N-channel MOSFETs Q5 and Q6, respectively. They ensure that the MOSFETs remain in the off state when there are no control signals from the Arduino's digital I/O pins.

**Fig. 5:**
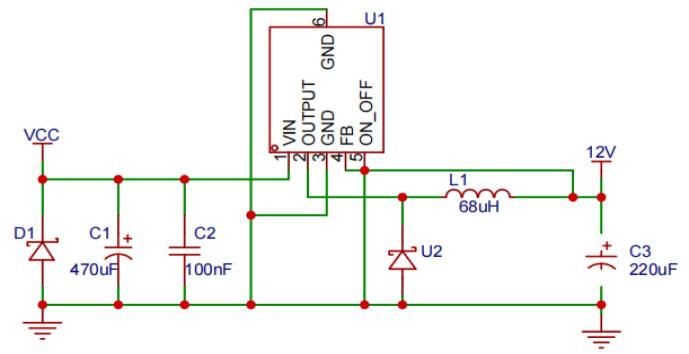
Schematic diagram of the 12 V regulated circuit.

Fig. 5 illustrates the 12 V regulated circuit used in the design. The external voltage input (VCC) comes from the output of a power bank, which provides an adjustable power supply. This input is regulated to a steady + 12 V using the LM2596（Texas Instruments, USA） step-down voltage regulator. The regulated 12 V output is used to power both the IR2104S driver chips and the cooling system fan. Components such as capacitors (C1, C2, C3) and inductor L1 are used to stabilize the voltage, reduce noise, and filter the output, ensuring smooth and efficient operation.

**Fig. 6:**
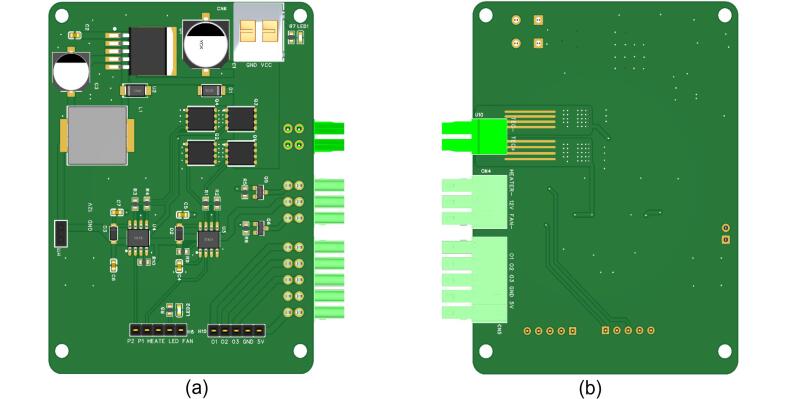
3D PCB model of the designed driver board (a) front side view and (b) back side view.

The driver board is designed to ensure stable operation of the Peltier module, fan, and heated lid, with a focus on efficient power regulation and reliable control signals. It integrates the H-bridge circuit from Fig. 3, the fan and heated lid control circuits from Fig. 4, and the voltage conversion circuit from Fig. 5. The 3D model of the driver board, shown in Fig. 6, provides a compact and efficient layout, displaying how all components are interconnected to meet the system's power and control needs.

### 2.3 PCR system power accessory

The PCR system uses the portable power bank (SC-1076, Guangzhou Longxing Electronics) as the primary power module. In addition, it is compatible with a 24  V DC power supply, providing a cost-effective alternative when using mains power.

With a battery capacity of 20,000 mAh and an energy rating of 74 Wh, the SC-1076 power bank provides stable and reliable continuous power. Its DC output ranges from 5  V to 24  V at up to 5 A, providing a maximum power output of 120  W, which fully meeting system requirements. Under a four-well load, the SC-1076 can support running a 72 min amplification (30 cycles) for a total of 2 runs on a single charge. In addition, the power bank is compatible with both USB-C and USB-A interfaces and supports fast charging technologies such as PD 3.0, PPS, and QC 3.0, thereby increasing the flexibility and adaptability of the system. The SC-1076 connects to the driver board via standard wiring and a 5.5  mm power output interface. It provides regulated 5  V and 12  V outputs via onboard voltage regulation units, which power critical components such as the controller, sensors, fan, and heated lid. Moreover, multiple protection mechanisms, such as over-voltage, over-current, and short-circuit protection, ensure safe and stable operation.

### 2.4 Piecewise variable coefficient PID algorithm for temperature control

PCR involves several vital temperature-controlled steps for amplifying DNA [25]. The process begins with pre-denaturation at 95 °C, where the double-stranded DNA is separated into single strands. The next step is denaturation, also at 95 °C, to continue separating the DNA strands for primer binding. Annealing follows at 55 °C, allowing primers to bind to the single-stranded DNA. Then, during the extension stage at 72 °C, Taq DNA polymerase synthesizes new DNA strands by adding nucleotides. Finally, a final extension at 72 °C ensures that any remaining single-stranded DNA is fully extended. Accurate temperature control is essential for successful PCR amplification. To achieve precise regulation across these stages, we implemented a piecewise variable coefficient PID [26] control algorithm. This algorithm manages temperature for each PCR step, including pre-denaturation, denaturation, annealing, extension, and final extension, with distinct temperature targets and PID control parameters. The control system regulates the heating block using a Peltier element, ensuring that the temperature is maintained within the required range for each stage.

**Fig. 7:**
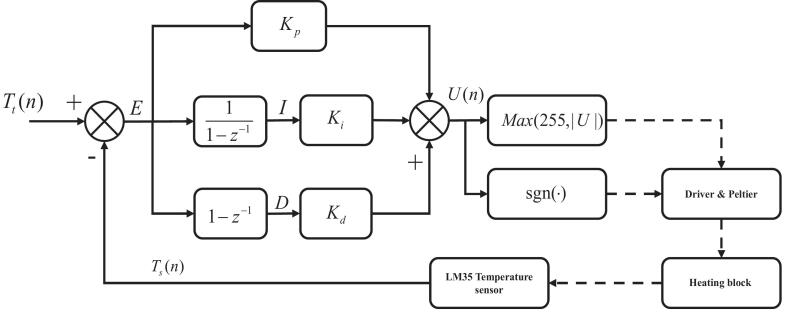
Schematic diagram of piecewise variable coefficient PID algorithm for PCR temperature control.

The PID control algorithm is shown in Fig. 7, which outputs control signal to regulate the temperature of the heating block via a Peltier element as follows:

Step1: temperature $T_{s}\left(n\right)$ feedback is obtained from the LM35 temperature sensor mounted on the heating block; The target temperature is $T_{t}\left(n\right)$.

Step2:$T_{t}\left(n\right)$ is compared with $T_{s}\left(n\right)$, generating an error signal $E\left(n\right)=T_{t}\left(n\right)-T_{s}\left(n\right)$. The integral of the error is denoted as I, $I=\sum {\;}_{i=0}^{n}E\left(i\right)$.The derivative of the error is D, D(n)=E(n)-E(n-1). Control parameters of the PID algorithm are denoted as Proportional $K_{p}$, Integral $K_{i}$, and Derivative $K_{d}$. Then the controller’s control signal U(n) can formular as: $U\left(n\right)=K_{p}\times E\left(n\right)+K_{i}\times I\left(n\right)+K_{d}\times D\left(n\right)$.

Step3**:** U(n) is constrained to a maximum value using the function Max(255,∣U∣) to ensure that the signal remains within the range of an 8-bit PWM signal. A sign function determines whether the system should heat or cool based on the direction of the U(n).

Finally, the control signal is sent to the driver board to control the Peltier element, which heats or cools the block to achieve and stabilize the desired temperature.

**Fig. 8:**
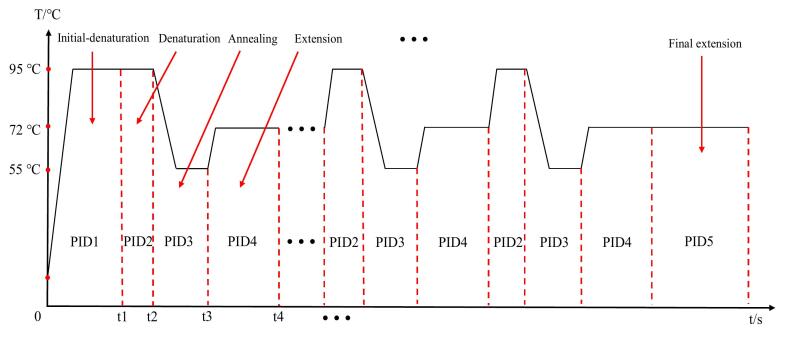
Chronology diagram of PID control algorithms and the correspond PCR stages.

Table 1 shows the control parameters optimized for the specific requirements of each PCR step as determined by manual fine-tuning using the Engineering Tuning method [27]. For initial-denaturation and denaturation (95 °C), the system uses PID1 and PID2 with parameters K_P_ = 15, K_i_ = 1, and K_d_ = 7. During the annealing stage at 55 °C, PID3 is applied with parameters K_p_ = 9, K_i_ = 0.1, and K_d_ = 5. For both extension and final extension at 72 °C, PID4 and PID5 are used with K_P_ = 13, K_i_ = 1, and K_d_ = 10. These parameters are optimized to meet the thermal requirements of each stage, minimizing overshoot and ensuring temperature stability. Fig. 8 illustrates the timing and sequence of the PID control algorithms during the PCR process, ensuring that the temperature is maintained at the desired levels for efficient DNA amplification. The denaturation, annealing, and extension stages are typically repeated for 25 – 35 cycles, allowing for the exponential amplification of the target DNA sequence.

**Table 1:** Coefficients of piecewise PID algorithm and the corresponded PCR stage.

**PCR stage**	**Algorithm**	**Target temperature (**°C**)**	**K_P_**	**K_i_**	**K_d_**
Initial-denaturation	PID1	95	15	1	7
Denaturation	PID2	95	15	1	7
Annealing	PID3	55	9	0.1	5
Extension	PID4	72	13	1	10
Final extension	PID5	72	13	1	10

### 2.5 Module assembly

**Fig. 9:**
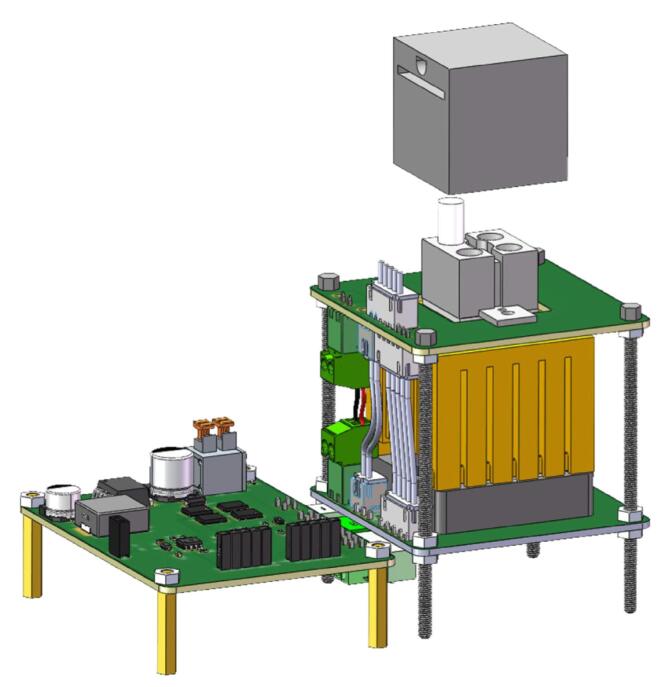
Assembled model of the PCR device.

Fig. 9 shows the assembled model of the system, which integrates the driver board and the PCR temperature control unit. PCB boards, copper pillars, and hexagon socket head cap are used to tighten and construct the mounting body of the instrument. These copper pillars and hexagon socket head cap screws ensure stability and alignment across multiple layers of the system. The connection between the driver board and the PCR temperature control unit is established through male and female connectors, enabling easy assembly and disassembly for maintenance or upgrades. This modular design approach provides a robust yet flexible platform for managing temperature control essential for PCR amplification.

## 3 Design files summary

The mechanical components of the PCR device, including the 4-well heating block, heated lid, heat sink, and the overall structure and assembly, were designed using SolidWorks, a powerful Computer-Aided Design (CAD) software commonly used for detailed 3D modeling and engineering designs. The circuit design and PCB layout were developed using JLCEDA Pro V2.2.31, an easy-to-use electronic design automation tool that is free for personal use. In addition, APP Designer in MATLAB was used to create the graphical user interface (GUI) of the PC software, which provides intuitive, user-friendly interaction with the algorithms for parameterization and temperature control. See (Table 2).

**Table 2:** Design file form.

**Folder Name**	**Design file name**	**File type**	**Open source license**
PCR device model design	PCR instrument prototype design	.SLDASM,.SLDPRT,.png	MIT License
	4-well aluminum heating block	.STL	
	Heated lid casing	.STL	
Circuit design	1Driver board circuit	.epro,.pdf	MIT License
	2Top support board	.epro,.pdf	
	3Bottom support board	.epro,.pdf	
PC software	app1	.mlapp	MIT License
	app_1	.m	

## 4 Bill of materials summary

Materials used in the system are categorized into two sections: circuit components and mechanical components. These are outlined in Table 3 and Table 4, respectively, providing a detailed list of the parts required for both the electronic and mechanical aspects of the assembly. These tables offer a clear breakdown of the components and their associated costs, ensuring transparency for the entire system's construction.

**Table 3:** Circuit components form.

**Designator**	**Component**	**Number**	**Cost per unit - USD**	**Total cost - USD**	**Source of materials**	**Material type**
C1	470uF	1	0.06	0.06	https://e.tb.cn/h.ToUKzgSSYVTjlXV	Non-specific
C2, C4, C6	100nF	3	0.01	0.03	https://e.tb.cn/h.To8tzS5t8AUyhLA	Non-specific
C3	220uF	1	0.041	0.041	https://e.tb.cn/h.ToUJuZWxrQlqhl8	Non-specific
C5, C7	1uF	2	0.01	0.02	https://e.tb.cn/h.TozqZ3txyUIXDpy	Non-specific
R1, R2, R3, R4	22R	4	0.01	0.02	https://e.tb.cn/h.ToKpoSvJqpo1WVJ	Non-specific
R5, R6, R9, R10	10K	4	0.01	0.04	https://e.tb.cn/h.To8FxBFJGd8JVVJ	Non-specific
R7	4.7K	1	0.01	0.01	https://e.tb.cn/h.To8Fa4cHKm6UhyO	Non-specific
R8	3.3K	1	0.01	0.01	https://e.tb.cn/h.ToKGfl7APCzdUgV	Non-specific
L1	68uH	1	0.42	0.42	https://e.tb.cn/h.ToKCLxHHCTGdHSq	Alloy inductors
CN4	JL15EDGA-35003G01	1	0.15	0.15	https://e.tb.cn/h.Toq1BDlnyeoNK69	Flame retardant nylon copper tin plated
CN5	JL15EDGA-35005G01	1	0.25	0.25	https://e.tb.cn/h.ToiIeeAzSpBEc0X	Flame retardant nylon copper tin plated
CN6	XY119A-5.0-2P	1	0.06	0.06	https://e.tb.cn/h.Toqd6p0c7Qlk0pI	Flame retardant nylon copper tin plated
D1	ES1G	1	0.10	0.10	https://e.tb.cn/h.ToUqmrv7pdM4Kaq	Non-specific
D2, D3	DSK14	2	0.02	0.04	https://e.tb.cn/h.ToOFzxcttX94CGj	Non-specific
H8, H10	2.54-1*5P	2	0.01	0.02	https://e.tb.cn/h.ToiGNbnlAO0LO7x	Non-specific
H11	2.54-1*2P	1	0.01	0.01	https://e.tb.cn/h.ToiGNbnlAO0LO7x	Non-specific
LED1, LED2	NCD0603R1	2	0.01	0.02	https://e.tb.cn/h.ToA5jKgwqGYwygI	Non-specific
Q1, Q2, Q3, Q4	BSC0702LS	4	0.53	2.12	https://e.tb.cn/h.ToqRjLhmrctB1ML	Non-specific
Q5, Q6	AO3400	2	0.28	0.56	https://e.tb.cn/h.TogFLn6Ynt49unL	Non-specific
U1	LM2596S-12	1	0.28	0.28	https://e.tb.cn/h.TovEzPtfU7Vkynq	Non-specific
U2	SS54	1	0.02	0.02	https://e.tb.cn/h.ToOHtEsgmMJuEK5	Non-specific
U3, U4	IR2104STRPBF	2	0.46	0.92	https://e.tb.cn/h.TojpaMEI9Rp6Ehe	Non-specific
U10	KF2EDGA-3.5-2P	1	0.20	0.20	https://e.tb.cn/h.TojLle8kSklJmVu	Flame retardant nylon copper tin plated
CN3	KF2EDGV-3.5-2P	1	0.06	0.06	https://e.tb.cn/h.ToAIuWWr9dBhQgP	Flame retardant nylon copper tin plated
U1, U3	BX-XH2.54-5PZZ	2	0.03	0.06	https://e.tb.cn/h.TojARutMZb3U0g6	Nylon Brass
U4	BX-XH2.54-2PZZ	1	0.01	0.01	https://e.tb.cn/h.ToAFnthepWBEYft	Nylon Brass
CN3	MX15EDGRC-3.5-05P-GN01-Cu-A	1	0.04	0.04	https://e.tb.cn/h.TojGRRa2EIys3ZK	Flame retardant nylon copper tin plated
CN6	KF2EDGV-3.5-2P	1	0.06	0.06	https://e.tb.cn/h.ToAIuWWr9dBhQgP	Flame retardant nylon copper tin plated
CN7	MX15EDGRC-3.5-02P-GN01-Cu-A	1	0.01	0.01	https://e.tb.cn/h.ToAw048RMiHHVMF	Flame retardant nylon copper tin plated
CN9	DB2ERC-3.5-3P-GN	1	0.02	0.02	https://e.tb.cn/h.ToAw048RMiHHVMF	Flame retardant nylon copper tin plated
U5	BX-XH2.54-5PZZ	1	0.3	0.03	https://e.tb.cn/h.TojARutMZb3U0g6	Nylon Brass
U6, U7	BX-XH2.54-2PZZ	2	0.01	0.02	https://e.tb.cn/h.ToAFnthepWBEYft	Nylon Brass
Temperature Sensor	LM35CAZ	2	0.51	1.02	https://e.tb.cn/h.ToRMIK0RAY8P0MC	Non-specific
Controller	Arduino UNO R3	1	4.27	4.27	https://e.tb.cn/h.ToRndnkr1aZ7TsX	Electronics
Power bank	SC-1076	1	40.33	40.33	https://e.tb.cn/h.TLa0vPnQt4uSZzZ	Electronics

**Table 4:** Mechanical components form.

**Designator**	**Component**	**Number**	**Cost per unit - USD**	**Total cost - USD**	**Source of materials**	**Material type**
Heat sink	40x38x35mm^3^	1	3.28	3.28	https://e.tb.cn/h.ToOMViIfxtzpmLc	Aluminum
Fan	4cm,3P and 4500rpm	1	2.14	2.14	https://e.tb.cn/h.ToOMViIfxtzpmLc	PBT
4-well aluminum heating block	3D Printed	1	21.37	21.37	http://myc3d.com/	Aluminum
Heated lid casing	3D Printed	1	27.07	27.07	http://myc3d.com/	Aluminum
Thermoelectric cooler	TES1-12703	1	9.69	9.69	https://e.tb.cn/h.ToU8Eyyx9V1ZUUY	Semiconductor
Heated lid	12V14W Dry firing surface temperature of about 220 °C	1	3.99	3.99	https://e.tb.cn/h.TogtaA9qyglSSWZ	96% Aluminum oxide
Hexagon Socket Head Cap Screws	M3*70	4	0.05	0.20	https://e.tb.cn/h.ToUl4pxjDH6GVST	304 stainless steel
Hexagonal Copper Column	M3*20+4	4	0.06	0.24	https://e.tb.cn/h.ToOqWIG03Rm6voG	copper
Hex nut	M3	12	0.01	0.12	https://e.tb.cn/h.ToUOdzalzP5eeEw	304 stainless steel
Power cable	5.5*2.1 Male Wire Gauge: 1.27 Square, Length: 50 CM	1	0.66	0.66	https://e.tb.cn/h.ToOrMgOSzaRitVJ	copper
Dupont wire	10cm，40pin Male to Female	9	0.01	0.09	https://e.tb.cn/h.TogttkOzSfAX0He	copper
Thermal grease	Thermally conductive silicone	1	0.39	0.39	https://e.tb.cn/h.ToUmhbDOs1ldcJP	Non-specific

Table 3 lists the circuit components, including the designator, specific component name, quantity, unit cost (in USD) and total cost for each part. Similarly, Table 4 focuses on the mechanical parts, including items such as heat sinks, structural supports and 3D printed components. The tables outline the unit cost and source for each mechanical part, listing material types and suppliers for easy reference. This ensures that all components can be easily sourced to enable efficient reproduction of the PCR device based on the open-source files provided.

## 5 Build instructions

### 5.1 Circuit and mechanical modules manufacturing

The manufacturing process for the driver board involves several key steps: circuit design, PCB fabrication, component soldering, assembly, and final testing. The 3D PCB model of the driver board is shown in Fig. 6 (for the schematic diagram, refer to Driver circuit schematic.pdf). The PCB was designed as a two-layer board using FR-4 material and manufactured by Shenzhen JLC Technology Group Co., Ltd. High-quality components, as listed in Table 3, were carefully selected and soldered onto the board. Precise control of soldering temperature and time was maintained to ensure reliable electrical performance and durable connections.

**Fig. 10:**
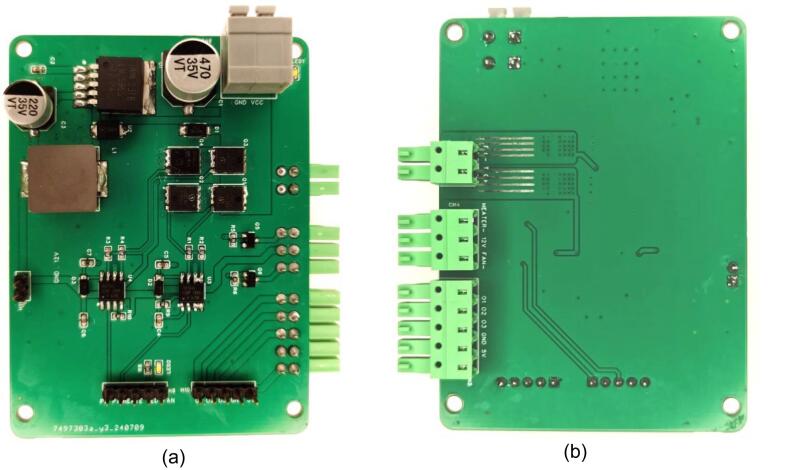
The front and back views of the manufactured driver circuit board (a) front side view and (b) back side view.

After assembly, the driver board underwent extensive debugging and testing. The functionality of each module, including MOSFETs, voltage regulators, and other key components, was verified to ensure proper circuit connectivity and input–output responses. The board was further tested under simulated working conditions, with a focus on the stability of the H-bridge driver's power output and the response speed of temperature adjustments. Fig. 10 shows the front and back views of the final manufactured driver board.

### 5.2 Top and bottom support boards manufacturing

**Fig. 11:**
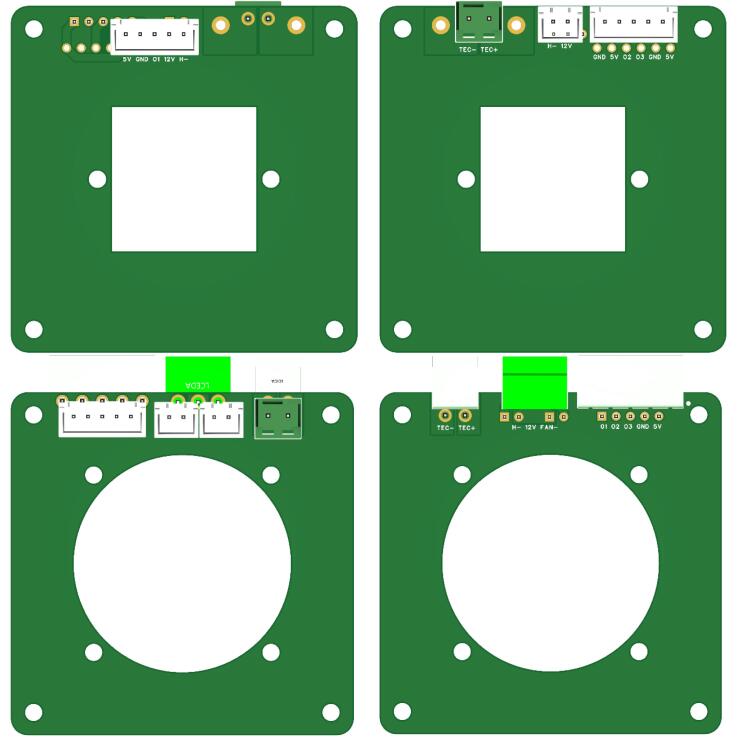
3D PCB models of top and bottom support boards.

Similarly, Fig. 11 shows the 3D PCB models of the top and bottom support boards. These boards were also fabricated using FR-4 material with 2 layers and a thickness of 1.2 mm to ensure structural support and reliability. The manufacturing process for the support boards followed the same steps as the driver board and will not be repeated here.

### 5.3 PCR temperature control unit construction

**Fig. 12:**
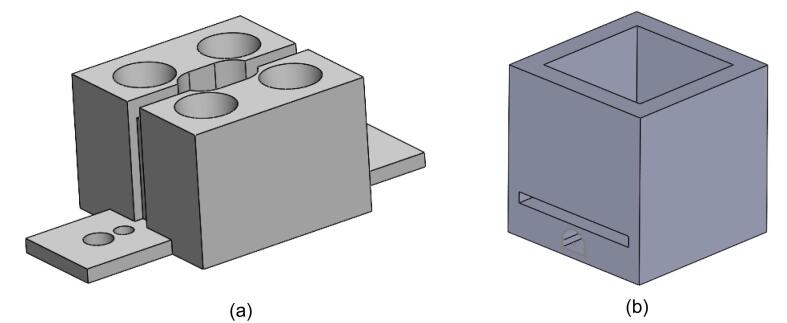
3D model of (a) 4-well aluminum heating block and (b) heated lid.

Fig. 12 shows the 3D models of the 4-well aluminum heating block and the heated lid. These components were fabricated using 3D printing, based on the provided design files located in the project folder. The relevant 3D files used for fabrication are “4-well aluminum heating block.STL” and “Heated lid casing.STL”. The aluminum alloy 3D printing service was provided by Hangzhou Meiyi Orange Design Co., Ltd [28].

### 5.4 Steps for PCR temperature control unit assembly

**Fig. 13:**
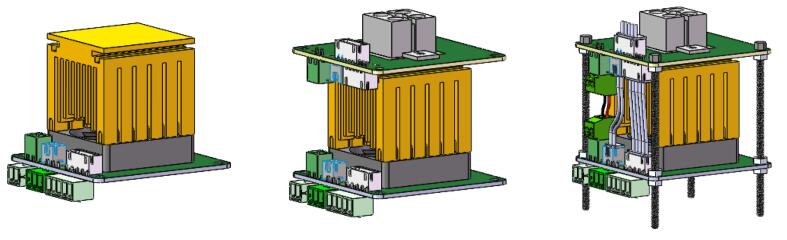
Sequence chart of PCR temperature control unit assembly.

Fig. 13 outlines the assembly steps for the PCR temperature control unit: first, attaching the fan to the bottom support plate and securing it with M3 screws. Then, solder the fan's circuit wires to the bottom board. Second, install the LM35 temperature sensor into the central temperature measurement hole of the 4-well aluminum heating block. To improve measurement accuracy, ensure any gaps are filled with thermal paste (Liyang Hongda Rubber Industry Co., Ltd.) to eliminate air pockets. The assembly sequence from top to bottom includes the 4-well aluminum heating block, the thermoelectric cooler (TES1-12703), the top support plate, and the heat sink, all secured together with M3 screws. Afterward, solder the thermoelectric cooler and the LM35 temperature sensor extension wires to the top support plate. Third, using long M3 screws (7 cm) and nuts, lock the partially assembled top and bottom support boards together, forming a unified structure. Connect the support boards with multiple XH2.54 cables.

Next, install the additional LM35 temperature sensor and the PTC ceramic of the heated lid into the appropriate slots: the LM35 sensor into the designated measurement hole and the PCT into the heating plate slot of the heated lid. Connect the external wires from both the sensor and the PTC ceramic of the heated lid to the XH2.54 cable connectors located on the top support plate. Once this step is completed, the PCR temperature control unit assembly is finished.

**Fig. 14:**
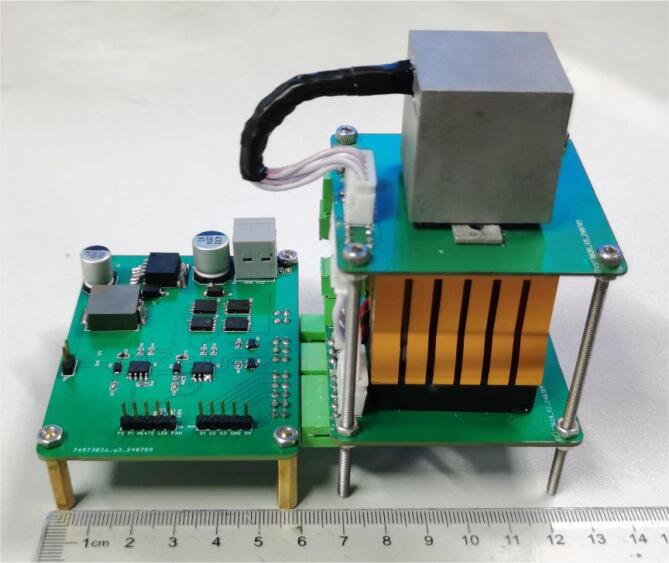
The prototype of the assembled PCR device.

Finally, check that the driver boards are properly aligned and securely connected. With this, the assembly of the entire PCR device is complete. A prototype of the assembled device is shown in Fig. 14.

### 5.5 Security measures in system implementation and operation

To ensure successful implementation and safe operation of the system, it is essential that proper safety precautions are taken during assembly, use, and handling of the components. Although the system is designed to minimize risk, users must follow the following guidelines to ensure personal safety, protect equipment, and maintain biosafety standards [29], [30] during PCR system operation.

## 6 Operation instructions

### 6.1 Software setup and operation instructions

The PC software requires a MATLAB environment to run. First, open the folder “APP Designer” and navigate to the file “app_1.m”, where you can adjust the PID parameters as needed. To start the GUI, you should open the file “app_1.mlapp”. From there, you can enter the desired temperature and time duration according to the specific stage of PCR amplification requirements.

### 6.2 Software interface instructions

We developed a user-friendly interface for controlling the PCR device using the MATLAB Support Package for Arduino. The entire codebase is packaged into an APP, enabling users to directly interact with the PCR system. Through the GUI, users can adjust key control parameters, set cycle counts, and specify target temperatures. In addition, the interface allows real-time monitoring of the temperature of both the heated lid and the heating block.

**Fig. 15:**
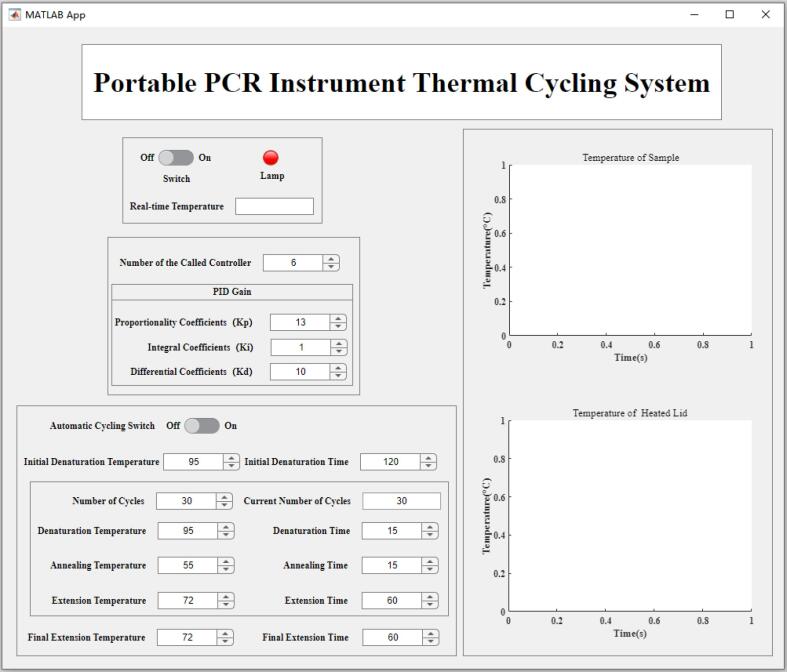
GUI of the proposed PCR system.

Fig. 15 shows the GUI of the proposed PCR system, which consists of four main sections:

(1) Title and main power switch.

At the top, the interface displays the system name, “Portable PCR Instrument Thermal Cycling System”, indicating that it is a temperature control system for a portable PCR instrument. At the top left is an “On/Off” toggle switch for power control and a “Lamp” indicator. Below, a “Real-Time Temperature” display shows the current temperature being monitored by the system.

(2) PID parameter setting.

The center of the interface displays the current PID controller number. The software determines which segment of the piecewise variable coefficient PID algorithm to apply based on the PCR target temperature and the real-time feedback temperature. When a PID algorithm is selected, its corresponding proportional (K_p_), integral (K_i_), and derivative (K_d_) coefficients are displayed below. These PID gains can also be set manually by the user.

(3) PCR cycles setting.

Users can configure the PCR cycling parameters with an “Automatic Cycling Switch” to enable or disable automatic cycling. The bottom section provides entry fields to set parameters such as number of cycles, initial denaturation temperature and time, denaturation temperature and time, and other relevant settings.

(4) Real-time temperature graphs.

On the right side of the GUI interface are two real-time temperature graphs labeled “Temperature of Sample” and “Temperature of Heated Lid”. These graphs plot temperature changes over time during the PCR reaction, allowing users to monitor the process in real time.

### 6.3 System operation instructions

**Fig. 16:**
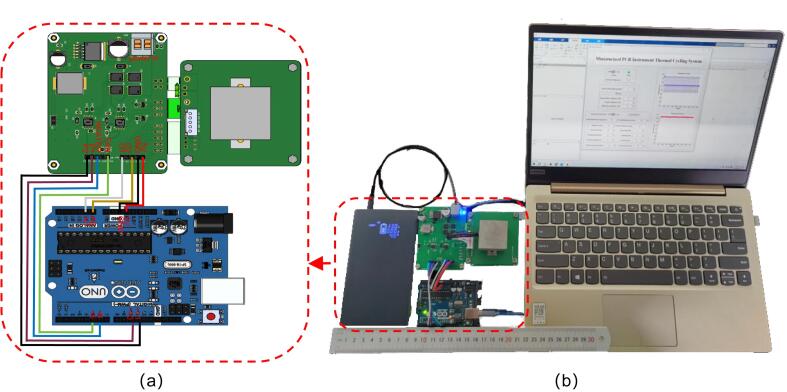
(a) Schematic diagram of the connection. (b)The prototype of the PCR system, including a power bank, the PCR device, an Arduino Uno controller, and a PC.

The complete setup of our prototype system is shown in Fig. 16. It measures 210 × 140 × 105 mm^3^ and weighs 670 g (not including the computer). To operate the system, follow these steps:

1. Connect the assembled PCR device to the Arduino UNO R3 using DuPont cables. The pin connections are as follows
• GND and 5 V terminals on the driver board are connected to the corresponding pins on the Arduino UNO R3.
• Temperature sensor outputs (o1 and o3) on the driver board are connected to analog input ports A0 and A1 on the Arduino.
• P2, P1, HEATE, and FAN on the driver board are connected to digital ports 11, 10, 6, and 5 on the Arduino, respectively.
2. Connect the power bank to the “power in” connector on the driver board using the power supply cable.
3. Connect the Arduino UNO R3 to the computer via a Type B USB cable.
4. Turn on the system, open the GUI interface, set the PCR parameters, and click the “On” button to start the system.

The complete Arduino and MATLAB GUI code is available at https://zenodo.org/records/14875968, where you can also find a short video demonstrating the device's operation.

## 7 Validation and characterization

### 7.1 Temperature calibration

Considering the temperature range from room temperature to nearly 100 °C, calibration is critical to ensure accurate readings when using the LM35 for PCR temperature measurement. Calibration over the full PCR temperature range was carried out using the water bath method. A waterproofed LM35 sensor was immersed in the water, and a high-precision mercury thermometer synchronously measure the actual temperature. The water bath was kept at a stable temperature for at least 10 min at each point to ensure temperature uniformity. The output voltage of the LM35 was recorded at each temperature, and a linear regression method was used to establish the calibration equation.

**Fig. 17:**
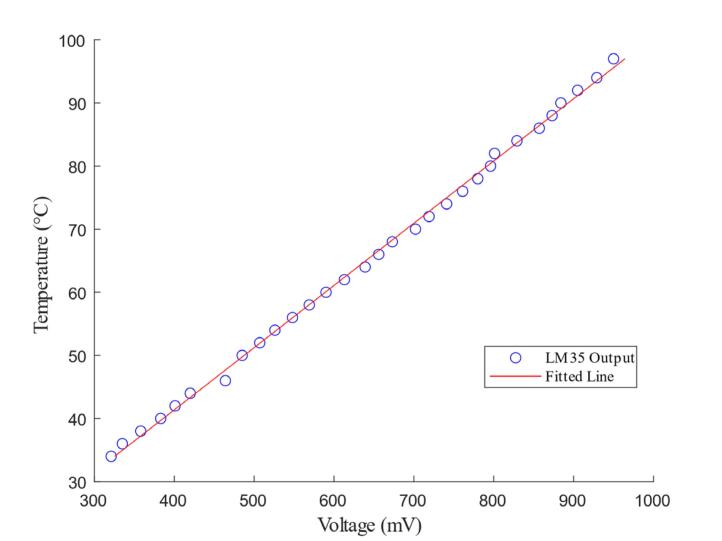
Measured temperature results and the corresponding calibration fit curve.

Fig. 17 shows the measured temperature data and the corresponding calibration fit curve. The LM35 sensor good linear behavior, and the calibration equation for the sensor is as follows:

T=(Vout+a)/b

where T (°C) is the temperature, and Vout (mV) is the output voltage of the sensor. The calibration parameters, *a* and *b*, were determined using the least squares method [31]. After the calibration process, the parameters of the sensor in the heating block were calculated as *a* = 19.50 and *b* = 10.14. With these calibration values, the sensor achieved a temperature measurement error of ± 0.55 °C, which meets the PCR instrument design requirement of maintaining accuracy within ± 1°C [32].

### 7.2 Heating block temperature control results

**Fig. 18:**
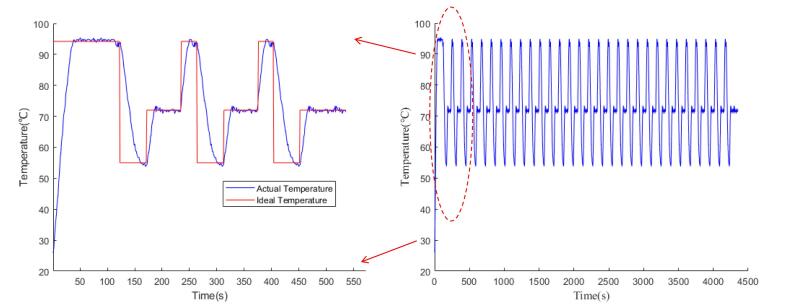
Heating block temperature curve over the entire PCR process.

A full PCR amplification thermal cycling test was conducted to evaluate the temperature control system's performance. Temperature variations during the process were recorded and plotted in Fig. 18. The control parameters were optimized for each stage of the PCR process: pre-denaturation at 95 °C, denaturation at 95 °C, annealing at 55 °C, and extension and final extension at 72 °C, following the specifications outlined in Table 1.

As shown in Fig. 18, once the system starts, the temperature rapidly rises from room temperature to 95 °C, marking the pre-denaturation phase. The temperature then cycles between the denaturation phase at 95 °C, annealing at 55 °C, and extension at 72 °C, repeating for the preset number of cycles, before the final extension phase completes the DNA amplification. During these temperature transitions, such as from 95 °C to 55 °C, the system quickly adjusts, with minimal overshoot and steady-state errors of less than 1°C. The heating and cooling rates remained consistent throughout multiple cycles, indicating good temperature stability of the system. Further analysis of the heating and cooling details showed that the initial heating phase started at 26.39 °C and reached the target temperature of 95 °C in 38 s, with an average heating rate of 1.78°C/s and a maximum rate of 2.8 °C/s. During the cooling phase, the temperature dropped from 94.83 °C to 55 °C in 26 s, with an average cooling rate of 1.52 °C /s and a maximum rate of 2.2 °C/s. These rates align well with the performance standards for PCR instruments, indicating that the designed system meets the necessary requirements.

**Fig. 19:**
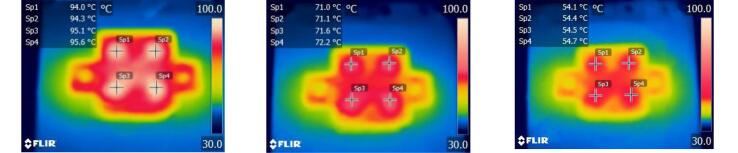
Infrared thermal images of the heating block at target temperatures of 95°C, 72°C, and 55°C.

To further verify the temperature control accuracy, a FLIR Ex-6 infrared camera was used to measure the heating block at target temperatures of 95 °C, 72 °C, and 55 °C, as shown in Fig. 19. The infrared images revealed good temperature uniformity across the four wells used for PCR tubes, with a temperature error of less than 1 °C compared to the target values. This slight discrepancy, larger than the LM35 sensor-calibrated error, may be due to the non-contact nature of infrared measurement, which has an inherent accuracy of ± 2 °C.

Overall, the experimental results demonstrate that the piecewise variable coefficient PID algorithm achieves precise temperature control, enabling rapid heating, cooling, and excellent temperature uniformity at each stage, meeting the thermal requirements for efficient PCR amplification.

### 7.3 Heated lid temperature

To prevent condensation of the reaction liquid on the PCR tube lid during the denaturation phase, where temperatures reach around 95 °C, our PCR device includes a heated lid that is maintained at 105 °C. Since slight deviations in temperature do not significantly impact the reaction, the heated lid does not require PID control feedback.

**Fig. 20:**
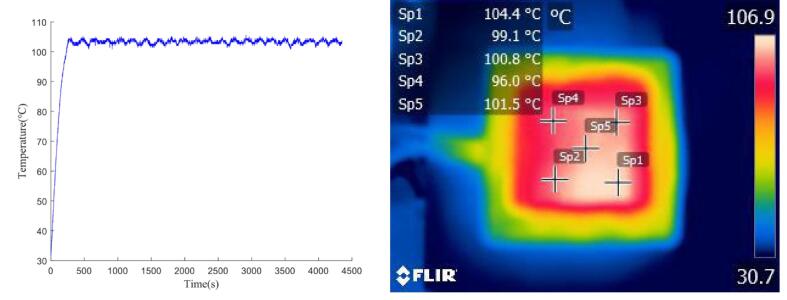
Temperature curve of the heated lid and its infrared thermal image at a target setting of 105 °C.

The real-time temperature data from the LM35 sensor and infrared thermal imaging, as shown in Fig. 20, confirm that the heated lid stabilizes around 105 °C, with a temperature error of ± 2 °C due to the absence of PID control and PWM regulation. Infrared imaging reveals a maximum temperature of 104.4 °C and a minimum of 96.0 °C, with higher temperatures near the PCR tube slots, consistent with expected heat dissipation patterns.

Overall, the heated lid effectively maintains a stable temperature of 105 °C, successfully preventing evaporation of the reaction solution and ensuring stable PCR conditions.

### 7.4 PCR amplification results

To validate the performance of our PCR prototype, kelp genes were amplified using both our prototype and a conventional commercial PCR instrument (ETC 811, Dongsheng) under identical conditions. The PCR protocol consisted of 30 cycles with the following parameters 95 °C for 15 s (denaturation), 55 °C for 15 s (annealing), and 72 °C for 1 min (extension). After thoroughly mixing the DNA fragments, the sample was evenly distributed into five tubes using a pipette (0.5 – 10 μL, eppendorf). One tube was processed using the commercial ETC 811 instrument, while the remaining four were amplified using our prototype. The amplification products were quantified using an ultra-micro nucleic acid quantification instrument (ND Onec, Gene Company Limited) and then analyzed by 1 % agarose gel electrophoresis (WIX-midiDNA, run at 135 V for 30 min) with SYBR Safe staining.

**Fig. 21:**
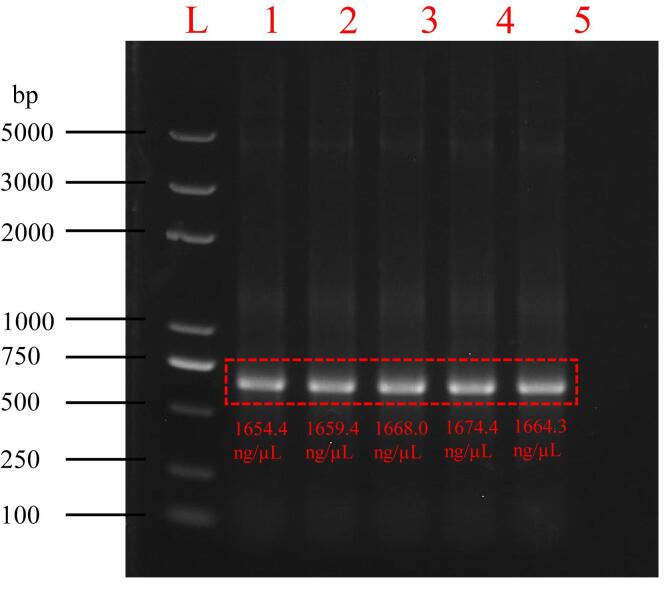
Electrophoresis results of kelp gene samples amplified by the proposed PCR device (columns “2-5″) and the ETC 811 PCR instrument (column ”1″). The DNA marker is labeled as “L”.

The final electrophoresis results are shown in Fig. 21. In the figure, the first lane labeled “L” represents a 5000 bp DNA marker used as a size reference. Lane 1 represents the amplification result from the commercial PCR instrument, while lanes 2–5 represent the results from our prototype. The measured concentrations of the PCR products for lanes 1–5 are also shown in the figure. Taking into account potential variations due to manual handling, instrument error, and the inherent exponential amplification characteristic of PCR [33], [34], our primary focus was on band position in the target region (highlighted by the red box in Fig. 21). All lanes show a single, distinct band at the expected ∼ 700 bp position, confirming successful target amplification. In addition, the brightness of the bands is consistent with the quantified product concentrations, 1654.4 ng/μL, 1659.4 ng/μL, 1668.0 ng/μL, 1674.4 ng/μL, and 1664.3 ng/μL for lanes 1 through 5, respectively. These results demonstrate that our PCR prototype effectively amplifies and detects DNA samples with performance comparable to that of a commercial PCR instrument.

## 8 Conclusion

We have developed a portable, low-cost PCR device that meets the needs of modern molecular diagnostics. Our prototype integrates a compact four-well thermal cycling system with a semiconductor-based temperature controller and an Arduino-driven piecewise PID algorithm that enables rapid temperature transitions with heating and cooling rates of 1.78 °C/s and 1.52 °C/s, respectively, while maintaining precise control within ± 0.55 °C. Measuring 210 × 140 × 105 mm^3^ and weighing 670 g, the instrument is powered by a power bank, making it ideal for resource-limited environments. Validated through kelp gene amplification experiments and benchmarked against conventional commercial instruments, the device offers significant advantages in terms of portability, ease of use and low cost (approximately $120). Its power bank operation further confirms its suitability for resource-limited and field settings, while its open-source design encourages further innovation by the scientific community. Future work will focus on comprehensive quantitative performance evaluations in different environments and the exploration of adaptive temperature control methods. We plan to investigate auto-tuning techniques, such as reinforcement learning-based approaches, to improve control efficiency and responsiveness.

## CRediT authorship contribution statement

**Kan Luo:** Writing – review & editing, Writing – original draft, Supervision, Software, Methodology, Conceptualization. **Wei Cheng:** Writing – original draft, Software, Methodology, Data curation. **Yu Chen:** Writing – review & editing, Writing – original draft, Validation, Methodology. **Qirong Zhang:** Writing – original draft, Visualization. **Chaobing Liang:** Validation, Software, Data curation. **Jianxing Li:** Writing – review & editing, Data curation. **Wu Wang:** Writing – review & editing, Methodology, Conceptualization.

## Declaration of competing interest

The authors declare that they have no known competing financial interests or personal relationships that could have appeared to influence the work reported in this paper.
